# Bilateral Accessory Head of First Lumbrical: Anatomical Insight and Surgical Implications during Carpal Tunnel Release

**DOI:** 10.15388/Amed.2026.33.1.15

**Published:** 2026-07-22

**Authors:** Anand Verma, Karan Kumar, Madhumita Patnaik, Manisha R. Gaikwad, Praveen Kumar Ravi

**Affiliations:** 1 All India Institute of Medical Sciences, Bhubaneswar, India

**Keywords:** accessory head, carpal tunnel syndrome, first lumbrical, papildomas raumens pluoštas, riešo kanalo sindromas, pirmasis sliekinis raumuo

## Abstract

Lumbrical muscles are essential for the coordination and precise movement of the hand; although anatomical variation in these muscles is rare, they can have significant clinical consequences, especially when they affect the carpal tunnel. Accessory heads of lumbrical are infrequent, typically unilateral, and often asymptomatic. However, when these variants pass through the carpal tunnel, they may decrease the tunnel’s volume, compress the median nerve, and contribute to the onset of Carpal Tunnel Syndrome (CTS). We present a rare case of a bilateral Accessory Head of the first Lumbrical Muscle (AHFL) discovered during a routine cadaveric dissection of a 65-year-old male. Recognizing such variations is crucial in clinical practice, as abnormal muscle tissue can complicate the diagnosis and surgical treatment of CTS. Preoperative imaging and thorough intraoperative examination can assist in identifying these variants, thus enhancing surgical planning and reducing complications.

## Abstract (it)

Sliekiniai raumenys yra būtini rankos koordinavimui ir tiksliam judėjimui; nors šių raumenų anatominiai pokyčiai yra reti, jie gali turėti reikšmingų klinikinių padarinių, ypač kai jie veikia riešo kanalą. Papildomi sliekinių raumenų pluoštai yra reti, paprastai vienpusiai ir dažnai besimptomiai. Tačiau, kai šie raumenų pluoštai kerta riešo kanalą, jie gali sumažinti šio kanalo tūrį, suspausti vidurinį nervą ir prisidėti prie riešo kanalo sindromo (CTS) atsiradimo. Pateikiame retą atvejį, kai 65 metų vyro lavono skrodimo metu buvo nustatyti abipusiai pirmojo sliekinio raumens papildomi pluoštai. Tokias variacijas atpažinti yra labai svarbu klinikinėje praktikoje, nes nenormalus raumenų audinys gali apsunkinti CTS diagnozę ir chirurginį gydymą. Priešoperacinė vaizdinė diagnostika ir išsamus intraoperacinis tyrimas gali padėti nustatyti šias variacijas, taip leistų pagerinti chirurginio gydymo planavimą ir sumažinti komplikacijų.

## Introduction

The lumbricals play a pivotal role in the intricate movements of the hand, as they are an integral component of the intrinsic muscles of the hand [1]. Anatomical variations in the lumbricals or any accessory muscle with or without vascular variation in the proximal aspect of the palm are clinically significant, as they increase the risk of developing carpal tunnel syndrome [2–4]. Accessory heads or additional muscle bellies of the lumbricals are rare, and, when present, they occur unilaterally and are usually asymptomatic [5,6]. Accessory heads of the lumbricals become clinically evident when they extend into the carpal tunnel, causing compression over the median nerve [7,8]. The decision to preserve or remove the accessory head of any muscle during carpal tunnel release remains controversial. This case report aims to present a rare bilateral accessory head of the first lumbrical muscle (AHFL) that passes through the carpal tunnel, thereby highlighting its clinical significance in carpal tunnel syndrome and the appropriate surgical management.

## Case Report

During a routine undergraduate cadaveric dissection of a 65-year-old male embalmed cadaver, we observed a rare bilateral presence of the AHFL extending from the radial side of the *Flexor Digitorum Superficialis* (FDS) to the tendon of the *first lumbrical* (L1) by crossing the carpal tunnel. The dissection was carried out following the guidelines described in Cunningham’s Dissection Manual. Upon reflection of the palmar aponeurosis, the AHFL muscle was identified bilaterally. On the right side, the proximal tendon was 4.71 mm in length and arose from the FDS muscle approximately 65 mm proximal to the carpal tunnel. The unipennate muscular belly of the AHFL on the right side measured 58.52 mm, which continued as a tendon (56.60 mm in length) distally in the carpal tunnel and merged with the tendon of L1 (which arises from the radial side of the first flexor digitorum profundus tendon) to form a dorsal digital expansion. On the left side, the course and attachment were similar, with a longer proximal tendon (19.61 mm in length) and unipennate muscular belly (80.92 mm), and a shorter distal tendon (13.62 mm). Therefore, the unipennate muscular belly of AHFL crosses the carpal tunnel in the left side, which increases the likelihood of developing carpal tunnel syndrome. Other structures in the forearm and hand were normal.

## Discussion

The first lumbrical (L1) muscle of the hand is a slender unipennate muscle that typically originates from the radial aspect of the tendons of the flexor digitorum profundus (FDP) of the index finger, within the palm distal to the carpal tunnel [1]. L1 runs radially along the metacarpophalangeal region and inserts into the dorsal digital expansion. Lumbricals function in conjunction with interosseous, thenar, and hypothenar muscles of the hand to generate the intricate and coordinated movements of the hand [9]. The primary functions of L1 are to flex the metacarpophalangeal joint of the index finger and extend both proximal and distal interphalangeal joints [10]. These movements are essential for the precision grip, manipulation of objects, and coordinated movements of the digits in daily activities such as writing, pinching, typing, and playing musical instruments. L1 is innervated by the branch of the median nerve and possesses a high density of muscle spindles, which provide proprioceptive feedback and fine motor control of the digits [10].

**Figure 1:**
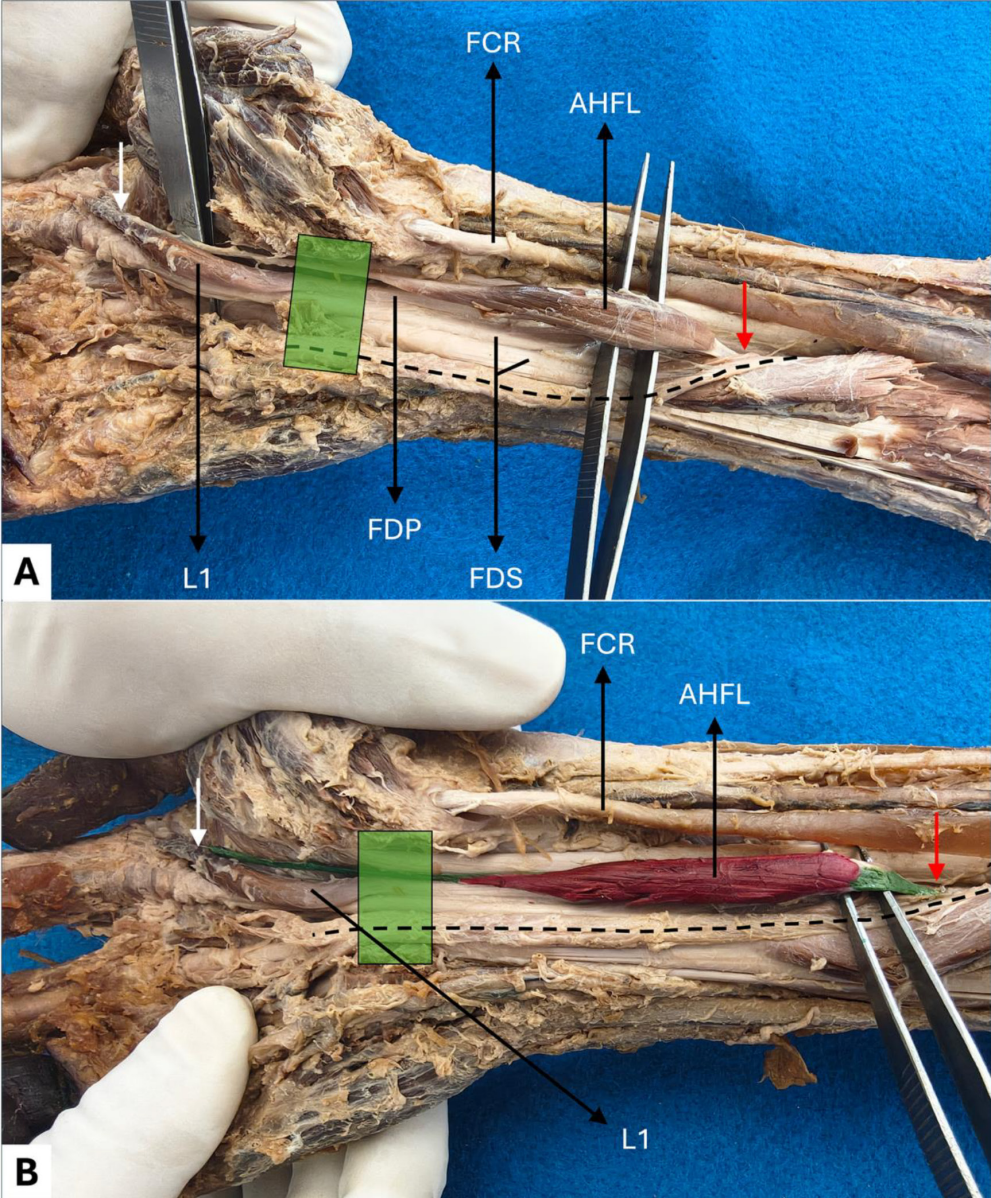
Accessory head of the first lumbrical (AHFL) in the right hand. (A) Uncolored and (B) colored dissection images show AHFL originating from the radial surface of the flexor digitorum superficialis (the red arrow) and joining the radial side of the first lumbrical (L1) (the white arrow) before its insertion. The muscular belly terminates proximal to the carpal tunnel. *Note*. FCR – Flexor carpi radialis; the black dotted line – reflected median nerve; the green rectangle – flexor retinaculum.

**Figure 2:**
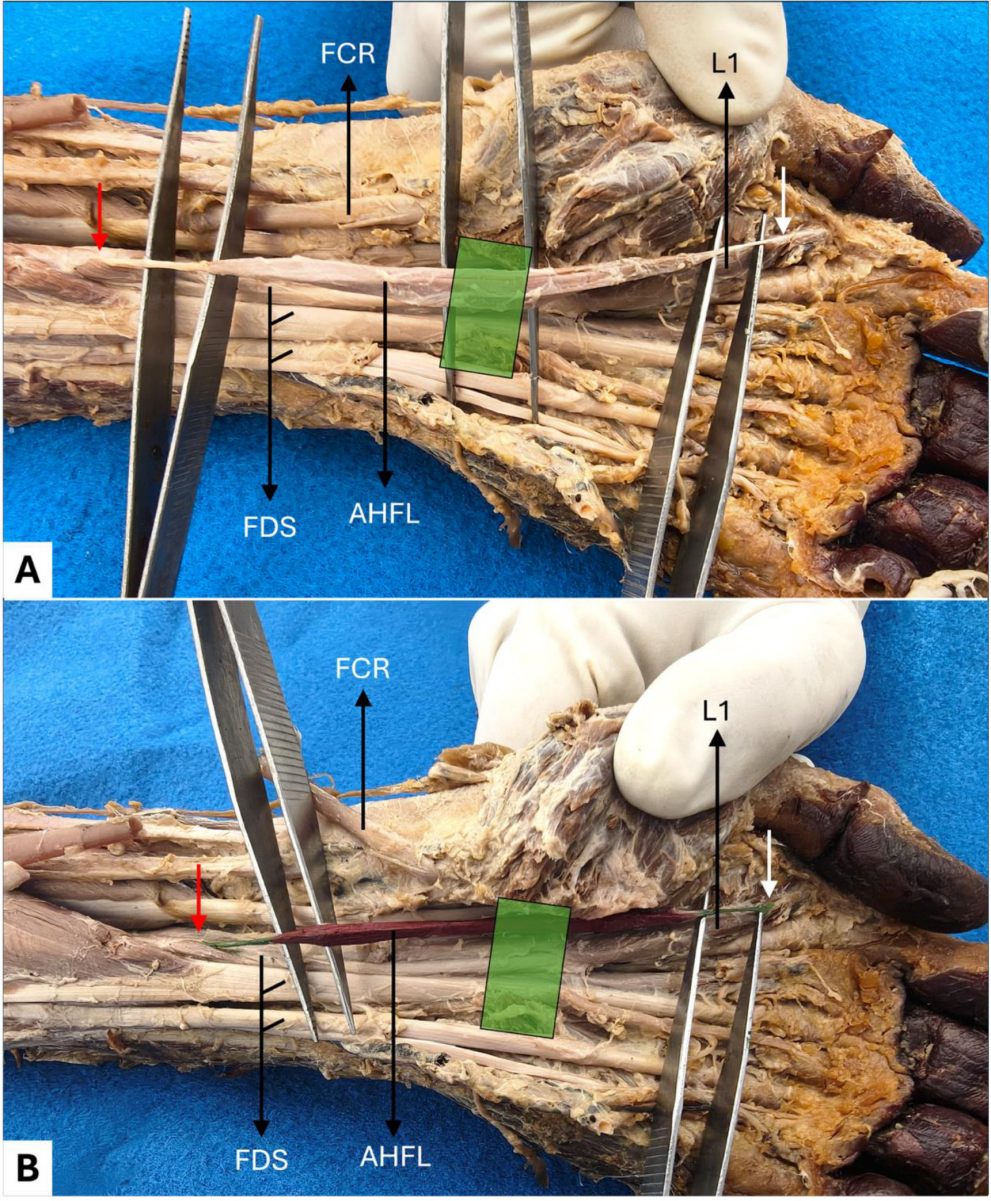
Accessory head of the first lumbrical (AHFL) in the left hand. (A) Uncolored and (B) colored dissection images show AHFL originating from the radial surface of the flexor digitorum superficialis (the red arrow) and joining the radial side of the first lumbrical (L1) (the white arrow) before its insertion. The muscular belly traverses the carpal tunnel. *Note*. FCR – Flexor carpi radialis; the green rectangle – flexor retinaculum.

Anatomical variations of the L1 muscle, including the accessory belly, supernumerary, or bifid origin, and proximal or anomalous origin, have been reported in the literature [11–14]. However, previous authors used different names for this variation. Since most cases reported with fusion of the accessory muscle belly or tendon with the normal lumbrical muscle, this variation is named as AHFL [6,11,15,16]. Limited literature exists on similar variations, and the incidence reported is from 2% to 5.41% unilaterally [11,14]. Bilateral AHFL is a very rare occurrence, and has only been reported as case reports (Table 1).

**Table 1:** List of accessory heads of first lumbricals (AHFL) reported in the literature along with its attachment, innervation, laterality and relation to carpal tunnel

Author (year)	Incidence of AHFL	Origin	Insertion	Innervation	Laterality	Relation to carpal tunnel
Afroze M (2000) [11]	5.41%	Radial side of FDS in the proximal forearm	Radial margin of L1 proximal to insertion	Median Nerve	Unilateral	Belly within/at carpal tunnel
Koizumi M (2002) [15]	Case report	Radial side, intermediate tendon of deep FDS	Radial margin of L1 proximal to insertion	Median Nerve	Bilateral	Right – belly pre-tunnel; Left – belly within/at carpal tunnel
Nayak S (2008) [6]	Case report	FDS tendon (index finger) at proximal retinaculum	Joins L1, both insert to DDS of index	Median Nerve	Unilateral	Belly within/at carpal tunnel
Sawant P (2015) [14]	2%	Radial side of most radial FDS tendon (index finger)	Radial side, dorsal digital expansion of index	Median Nerve	Unilateral	Post tunnel
Nam T (2024) [16]	Case Report	Radial side of second FDS muscle (distal forearm)	Merges with first lumbrical muscle	Not specified	Bilateral	Belly within/at carpal tunnel
Present Case	Case Report	FDS muscle belly origin ~65 mm proximal to carpal tunnel	Merges with L1 tendon into dorsal digital expansion	Median Nerve	Bilateral	Right – belly pre-tunnel; Left – belly within/at carpal tunnel

The present study reports a case of bilateral AHFL with the muscle belly traversing the carpal tunnel. This occurrence is clinically significant for diagnosing carpal tunnel syndrome (CTS) or planning for carpal tunnel release. The presence of muscular tissue within the carpal tunnel significantly reduces the available space. The normal carpal tunnel volume is approximately 5.51 cm^3^. A slight reduction in volume to 5.27 cm^3^ leads to mild CTS. However, when the volume decreases to 4.43 cm^3^, the individual experiences severe CTS [17]. Therefore, the presence of an additional muscle belly in the tunnel occupies a significant proportion of the available space, leading to the compression of the median nerve; this intensifies the symptoms of CTS. During the *Carpal Tunnel Release* (CTR), it may complicate the surgery and lead to a conversion of endoscopic CTR to the open procedure. Available evidence indicates that the removal of the accessory head of the lumbrical muscle head should be considered solely if it is directly responsible for nerve compression and persistent symptoms [7,18]. Conversely, preservation is preferred so that to prevent unnecessary loss of hand function. Selective excision typically results in symptom relief without substantial impairments in finger movement or grip strength, provided that the remaining intrinsic muscles remain intact [16,18].

Embryologically, myogenic cells from the ventral muscle mass differentiate into extrinsic and intrinsic muscle groups [19]. Incomplete separation or persistent connections between these precursor groups can result in the development of accessory muscle bellies connecting two groups [20]. Phylogenetic and comparative anatomical studies support this hypothesis, suggesting that the FDS and intrinsic hand muscles likely evolved from a shared ancestral flexor system. The bilateral occurrence seen in the present case may suggest a genetic predisposition or a symmetric developmental event during early limb bud formation [21]. One limitation of this case report is that it is a cadaveric study, and therefore it is impossible to report on the functional aspect of the accessory head of the first lumbrical. However, based on the literature, there is no functional loss of the finger movement or grip when the accessory head is removed. Consequently, its contribution to movement will be minimal, and its removal should be considered only when explicitly necessary [16,18].

## Conclusion

This case report documents a rare bilateral variation of the AHFL with its detailed morphometric anatomical description and its correlation to the clinical and embryological significance. The bilateral presentation is rare and denoted by asymmetric morphological characteristics. It demonstrates the complex nature of these developmental variations. Anomalous muscle tissue in the carpal tunnel may contribute to carpal tunnel syndrome and complicate surgical procedures. Preoperative imaging can help identify these variants and optimize surgical planning so that to avoid intraoperative and post-operative complication.
